# Nanopore Sequencing Technology Reveals the Transcriptional Expression Characteristics of Male Pig’s Testes Before and After Sexual Maturity

**DOI:** 10.3390/genes17010021

**Published:** 2025-12-26

**Authors:** Yiting Yang, Siyu Chen, Ziling Hao, Taizeng Zhou, Songquan Guan, Ya Tan, Yan Wang, Xiaofeng Zhou, Lei Chen, Ye Zhao, Linyuan Shen, Li Zhu, Mailin Gan

**Affiliations:** 1 Farm Animal Germplasm Resources and Biotech Breeding Key Laboratory of Sichuan Province, Sichuan Agricultural University, Chengdu 611130, China; yangyiting0914@stu.sicau.edu.cn (Y.Y.); 2023202015@stu.sicau.edu.cn (S.C.); 2022002001@stu.sicau.edu.cn (Z.H.); 202100425@stu.sicau.edu.cn (T.Z.); 18939294200@163.com (S.G.); tanya_lee@126.com (Y.T.); wangyan2023@sicau.edu.cn (Y.W.); zxf93715@163.com (X.Z.); chenlei815918@sicau.edu.cn (L.C.); zhye@sicau.edu.cn (Y.Z.); shenlinyuan@sicau.edu.cn (L.S.); 2 State Key Laboratory of Swine and Poultry Breeding Industry, Sichuan Agricultural University, Chengdu 611130, China; 3 Key Laboratory of Livestock and Poultry Multi-Omics, Ministry of Agriculture and Rural Affairs, College of Animal and Technology, Sichuan Agricultural University, Chengdu 611130, China; 4 Institute of Animal Husbandry and Veterinary, Guizhou Academy of Agricultural Science, Guiyang 550005, China

**Keywords:** nanopore sequencing, pig testis, sexual maturity, spermatogenesis, lncRNA, circRNA

## Abstract

**Background**: Testicular development and spermatogenesis are intricate biological processes controlled by a coordinated transcriptional network. However, comprehensive characterization of full-length transcripts and non-coding RNAs (ncRNAs) during porcine testicular sexual maturation remains limited. **Methods**: This study systematically profiled the transcriptional landscape of pig testes prior to (pre-sexual maturity, PSM) and following (post-sexual maturity, SM) sexual maturity using Oxford Nanopore Technologies (ONT) long-read sequencing. **Results**: There were 11,060 differentially expressed mRNAs (DEGs), 15,338 differentially expressed transcripts (DETs), 688 differentially expressed lncRNAs (DELs), and 19 differentially expressed circRNAs (DEcircRNAs) between PSM and SM groups among the 9941 mRNAs, 15,339 transcripts, 4136 lncRNAs (58.58% being LincRNAs). These differential RNAs converged on 133 shared GO terms (e.g., spermatogenesis, male gamete generation) and 58 common KEGG pathways (e.g., metabolic pathways, Wnt/MAPK signaling), according to functional enrichment and combined analysis. Core genes (e.g., *PRM1*, *ODF2*, *GSTM3*) demonstrated synergistic expression across gene, transcript, lncRNA-cistarget, and circRNA levels. Furthermore, DELs were associated with steroid biosynthesis and N-glycan biosynthesis, whereas DEcircRNAs, which were mostly upregulated after puberty, were thought to control genes linked to spermatogenesis. **Conclusions**: This research sheds light on the dynamic transcriptional reprogramming that occurs during the maturation of pig testicles, advances our knowledge of coding and ncRNA regulatory networks in male mammals, and offers useful molecular markers for enhancing pig reproductive efficiency.

## 1. Introduction

Mammalian sexual maturity onset constitutes a pivotal biological transformation, tightly regulated by the intricate crosstalk within the hypothalamic–pituitary–gonadal axis [1]. Gonadal functions are activated as a result of this neuroendocrine cascade, allowing the person to become reproductively competent. In addition to being of academic interest, the timeline and development of this event are inextricably linked to agricultural productivity, species fitness, and, in a wider translational context, provide insights into reproductive health and disorders [2,3,4]. One of the best models for studying the complexities of mammalian reproduction is the domestic pig (*Sus scrofa domestica*). Its physiological similarities to humans, including comparable organ sizes, metabolic traits, and, pertinently, reproductive cyclicity and testicular architecture, render it an invaluable biomedical model [5,6]. Furthermore, as the world’s second most consumed meat globally, pork’s production and consumption chain hinges heavily on boars. In the field of animal husbandry, testicular development and semen quality are direct determinants of production efficiency. Improving semen quality and accelerating sexual maturity can have significant financial advantages [7,8,9]. Therefore, clarifying the molecular underpinnings of pig sexual maturity is therefore crucial from both an economic and scientific standpoint.

Central to male reproductive maturation is the development and functional maturation of the testes. These organs are tasked with two primary functions: steroidogenesis, primarily the production of testosterone by Leydig cells [10], and spermatogenesis, the complex, multi-stage process of sperm production occurring within the seminiferous tubules, supported by Sertoli cells [11,12]. The period surrounding sexual maturity is characterized by a dramatic remodeling of the testicular landscape. Pre-pubertally, the testes are relatively quiescent, with gonocytes differentiating into spermatogonial stem cells [13]. The pubertal transition triggers a wave of cellular differentiation and proliferation: Sertoli cells cease mitotic activity and form the blood-testis barrier, Leydig cells mature to produce copious amounts of testosterone, and the spermatogenic lineage advances from spermatogonia through meiosis to the eventual production of haploid spermatozoa [14]. While the histomorphological changes during this period are well-charted, the comprehensive transcriptional program that drives and coordinates these events remains only partially decoded. The complete repertoire of messenger RNAs (mRNAs), with their diverse alternative splicing isoforms, and the regulatory roles of various non-coding RNA families during this critical window in the pig, are yet to be fully elucidated, representing a significant knowledge gap in reproductive biology.

With the advent of the multi-omics era, high-throughput sequencing has revolutionized the investigation of gene transcriptional profiles. The analysis of gene expression profiles in many biological contexts, including the testes, has been made possible by second-generation sequencing technology, which is based on short-read sequencing [15,16]. However, its inherent limitation lies in the fragmentation of transcripts, which poses substantial challenges for accurately reconstructing full-length splice variants, pinpointing transcription start and end sites, and confidently identifying complex non-coding RNA categories, including long non-coding RNAs (lncRNAs) and circular RNAs (circRNAs), which often have overlapping sequences with their linear counterparts or other genes [17]. The advent of third-generation sequencing platforms, notably Oxford Nanopore Technologies’ Direct RNA Sequencing (DRS), has heralded a new era by sequencing single, native RNA molecules in real time without relying on PCR amplification or reverse transcription [18,19]. This methodology offers several transformative advantages: it provides direct, full-length reads that encompass the entire transcript, enabling the precise definition of splice variants and the discovery of novel genes; it allows for the direct detection of RNA base modifications (the “epitranscriptome”); and it vastly simplifies the accurate identification and quantification of LncRNAs and CircRNAs. While nascent, the application of this powerful technology in reproductive biology is beginning to yield insights. For instance, recent studies in mice, pig and humans have used long-read sequencing to reveal previously unannotated testis-specific transcript isoforms [20,21]. Nevertheless, a comprehensive application of Nanopore DRS to simultaneously profile the dynamic expression of mRNAs, transcript isoforms, LncRNAs, and CircRNAs in the porcine testis across the critical threshold of sexual maturity has not been previously undertaken.

In summary, to better elucidate the gene-driven dynamics underlying male animal maturation, we employed the Oxford Nanopore Direct RNA Sequencing platform to address the previous technical limitations in such studies. Meanwhile, utilizing pigs as an excellent biological model enables more in-depth exploration of the genetic mechanisms governing male reproduction. Building on these objectives, this study aims to go beyond a mere list of gene expressions and provide an integrated, multifaceted biological analysis. We focused on the mRNA expression levels of transcriptional information and the expression dynamics of various non-coding RNAs. This research elucidates the complex transcriptional expression characteristics during testicular maturation, provides valuable high-resolution genomic resources, and delivers fresh perspectives on the molecular regulatory pathways underlying sexual maturation in major mammalian model systems.

## 2. Materials and Methods

### 2.1. Animals and Treatment

This research scheme was approved by the Sichuan Agricultural University Institutional Animal Care and Use Committee (Chengdu, China, No. 20240638, Approval date: 20 November 2024). This study selected six F1 generation individuals from Tibetan and Yorkshire pigs raised under the same feeding conditions and environmental control. The six TY crossbred pigs were divided into two groups based on age: Group 1 used the weaning period (28 days old) as the pre-sexual maturity group (PSM Group), and Group 2 used the fattening period (210 days old) as the post-sexual maturity group (SM Group). Following slaughter, we promptly isolated testicular tissue blocks for follow-up experiments, while samples designated for RNA sequencing were immediately stored in liquid nitrogen for preservation.

### 2.2. Hematoxylin-Eosin (HE) Staining Analysis

Fresh testicular tissue samples were fixed with a general-purpose tissue fixative and then dehydrated using a DIAPATH Donatello dehydrator (DIAPATH S.p.A., Martinengo, Italy) in a gradient manner before being embedded in paraffin. The embedded blocks were then prepared into paraffin sections using a Leica RM2016 pathological microtome (Leica Microsystems (Shanghai) Co., Ltd., Shanghai, China). The staining procedure was as follows: sections were dewaxed sequentially with environmentally friendly dewaxing solutions I and II for 20 min each, rehydrated, and treated with high-resolution constant staining pretreatment solution for 1 min; hematoxylin staining for 3–5 min, followed by differentiation with differentiation solution, bluing with a blue solution, and rinsing with running water; dehydration with 95% ethanol for 1 min, and staining with eosin for 15 s; subsequently, they were dehydrated and cleared sequentially with anhydrous ethanol, n-butanol, and xylene in a gradient manner, and finally mounted with neutral resin. The stained sections were observed for histological morphology using a Nikon ECLIPSE E100 upright optical microscope (Nikon Corporation, Tokyo, Japan), and the entire section was digitized and image analyzed using a Zhiyue WS-10 slide scanning system.

### 2.3. Scanning Electron Microscopy (SEM) Analysis

Fresh testicular tissue was harvested and trimmed into tissue slides ≤ 3 mm^2^. After gentle rinsing with PBS, the slides were fixed in SEM fixative (Servicebio, G1102, Wuhan, China) for 2 h, then transfer from room temperature to 4 °C for storage. After rinsing with 0.1 M PB, the samples were fixed with 1% osmium tetroxide at room temperature in the dark for 1–2 h, followed by gradient dehydration with 30–100% ethanol (15 min per step), and then replaced with isoamyl acetate for 15 min. After critical point drying, the samples were mounted on conductive carbon adhesive and sputtered with gold for approximately 30 s to enhance conductivity. Finally, the surface morphology of the samples was observed and images were acquired under a Hitachi SU8100 scanning electron microscope (Hitachi High-Tech Corporation, Tokyo, Japan). The image data were processed and analyzed using the accompanying Regulus SU8100 software.

### 2.4. RNA Extraction and Library Construction

We extracted total RNA from animal soft tissues with the R6834 Total RNA Kit I (Omega Bio Tek, Inc., Norcross, Norcross, GA, USA). Briefly, 15–30 mg of tissue was pulverized in liquid nitrogen and homogenized in TRK Lysis Buffer. The lysate was then passed through a homogenization column by centrifugation at 14,000× *g* for 2 min at room temperature to remove insoluble debris. An equal volume of 70% ethanol was added to the filtrate, and the mixture was applied to an RNA binding column. Wash the column sequentially with 500 µL RNA wash buffer I, centrifuge at 10,000× *g* for 30 s, discard the filtrate, then wash twice with 500 µL RNA wash buffer II, centrifuge at 10,000× *g* for 1 min, discard the filtrate. Finally, place the RNA binding column back into the collection tube and centrifuge at 10,000× *g* for 2 min. Transfer the RNA binding column to a new 1.5 mL centrifuge tube, add 30–70 µL of nuclease-free water, and centrifuge at 10,000× *g* for 2 min to elute the RNA. We evaluated the quality and concentration of RNA with a NanoDrop One spectrophotometer (Thermo Fisher Scientific, Waltham, MA, USA) and a Qubit 3.0 Fluorometer paired with the Qubit RNA Assay Kit (Life Technologies, Carlsbad, CA, USA), respectively. The Direct RNA Sequencing (DRS) library was prepared using the SQK-RNA004 kit (Oxford Nanopore Technologies, Oxford, UK). Briefly, 25 µg of total RNA was used as input, and poly(A)+ mRNA was enriched. The enriched mRNA was then ligated to a reverse transcription adapter using T4 DNA Ligase. Subsequently, reverse transcription was performed to synthesize the first-strand cDNA using reverse transcriptase. The resulting cDNA product was purified using Agencourt RNAClean XP beads. A sequencing adapter was ligated to the purified cDNA in a second T4 DNA Ligase reaction, followed by another bead-based purification step. The final library was prepared by mixing the adapter-ligated product with Sequencing Buffer and Library Solution. The prepared library was loaded onto a FLO-PRO004RA flow cell and sequenced on a PromethION sequencer (Oxford Nanopore Technologies, Oxford, UK) for 48 to 72 h.

### 2.5. Sequencing Data Pre-Processing

The raw output data from the Nanopore sequencer adopts the pod5/fast5 format, which encapsulates all original sequencing signals. We performed base calling using Dorado (version 0.9.0) [22] and converted into fastq sequence files. Given that raw sequencing data might include low-quality reads, we categorized the raw data into pass (Q ≥ 10) and fail (Q < 10) groups based on the average read quality during base calling to guarantee the credibility of subsequent analytical results. Data in the pass category were regarded as clean reads for subsequent analyses, and these filtered sequences were aligned to the reference genome via minimap2 [23] (version 2.17-r941). Alignment statistics were generated using samtools [24] (version 1.11) (Table 1). Transcript expression was quantified using salmon [25] (version 1.4.0). TPM calculations were performed by dividing the read count of each gene/transcript by its length (in kilobases) to obtain the reads per kilobase (RPK) of the gene/transcript. All RPK values were calculated for each sample and then divided by 1,000,000 to obtain a scaling factor per million. The RPK values were then divided by the scaling factor per million to obtain the TPM value.

### 2.6. Transcript Analysis and Identification

We utilized Flair software (version 1.5.0) [26] to acquire concordant sequences using alignment results. First, we transformed the reference genome’s bam file into bed12 format via the bam2Bed12.py script. Then, the identify_gene_isoform.py program was used to add gene or transcript information or alignment to chromosome position information. Finally, the collapse program was used for clustering and redundancy removal to obtain concordant sequences. We aligned the resulting concordant sequences to the reference genome and conducted transcript reconstruction with StringTie software (version 2.1.4) [27] based on the alignment results. StringTie’s merge mode was used to remove redundancy from the reconstructed transcripts, obtaining non-redundant transcript sequences. Coding region sequence prediction was performed on the newly identified transcripts. TransDecoder (https://github.com/TransDecoder/TransDecoder, accessed on 18 October 2018, version 5.5.0) serves as a widely employed CDS prediction tool that detects credible CDS from transcript sequences by leveraging features including ORF length and log-likelihood score. For the acquisition of comprehensive functional information regarding the novel transcripts, we conducted functional annotation of the transcripts with GO, KEGG, Nr, Uniprot, KOG/COG, Pfam, and PATHWAY databases.

### 2.7. Identification of LncRNAs

LncRNAs (long non-coding RNAs) refer to a category of RNA molecules featuring transcript lengths over 200 nucleotides (nt) and lacking protein-coding capabilities. The coding potential of newly identified transcripts was predicted using CNCI [28] (version 2.0), CPC2 [29] (v1.0.1), and PLEK software [30] (v1.2). The CPC2 tool conducts BLAST (v2.15.0) comparisons between transcripts and known protein databases, and evaluates the coding potential of transcripts by leveraging biosequence features of each coding frame through a support vector machine classifier. CNCI efficiently discriminates between coding and non-coding sequences according to adjacent trinucleotide frequency, and can reliably predict the coding potential of incomplete transcript pairs and antisense transcripts. PLEK software differentiates coding from non-coding transcripts based on the kmer composition of the sequence.

### 2.8. Identification of CircRNAs

The CIRI-Long (v1.1.0) [31] was used to detect circRNAs from sequencing data on the Nanopore platform. In short, the clean data was converted to FA format using seqkit software (v2.2.0), followed by circRNA detection for each sample using the CIRI-Long call module. Finally, quantification was performed using the CIRI-Long collapse module, and the bedgraph was converted using IGV software (v2.16.0) for visualization.

### 2.9. Gene Differential Analysis

The EdgeR (v3.32.1) software [32] was used for differential analysis of mRNAs, transcripts, lncRNAs, and circRNAs. First, the CPM value was calculated based on the reads_count data to filter genes/transcripts with low expression levels. Then, the reads_count expression matrix of the filtered genes/transcripts was standardized: (1) The logarithm of the reads_count values was taken, with 0 values still recorded as 0; (2) The mean expression level of each gene/transcript in the sample was calculated; (3) The mean expression level of each gene/transcript was subtracted from the corresponding mean expression level, calculated sequentially for each sample; (4) The median was taken for each sample; (5) The median was exponentially transformed to obtain the normalization coefficient sizefactor; (6) The original reads_count value was divided by the sizefactor of the corresponding sample to obtain the standardized expression level. Then, the average value (basemean) was calculated for each sample group of the standardized data. Dividing the basemean values of the two groups yielded the fold change (FoldChange). Finally, the negative binomial (NB) model was used to estimate the discrete values and calculate the *p*-value. After multiple testing and correction using the Benjamini–Hochberg (BH) method, the q-value was obtained. Differentially expressed genes were screened using *p*-value < 0.05 and |log2(FoldChange)| > 1 for enrichment analysis.

### 2.10. Functional Enrichment Analysis

For functional enrichment analysis, we conducted Kyoto Encyclopedia of Genes and Genomes (KEGG) enrichment analysis for differentially expressed genes via clusterProfiler (version 3.14.3) [33], while Gene Ontology (GO) enrichment analysis was carried out with topGO [34]. After ranking all genes according to their differential expression levels in the two groups, Gene Set Enrichment Analysis (GSEA) enrichment analysis and GSEA network construction were was plotted by https://www.bioinformatics.com.cn (last accessed on 10 December 2024), an online platform for data analysis and visualization.

### 2.11. Real-Time qPCR

We added 1 mL TRIzol reagent to 30 mg of testicular tissue and isolated total RNA following the kit’s operational guidelines. We assessed the concentration and quality of the extracted RNA with a NanoDrop spectrophotometer (Thermo Scientific, Waltham, MA, USA), then reverse-transcribed the total RNA into cDNA using the PrimeScript™ FAST RT reagent Kit with gDNA Eraser (TaKaRa, Dalian, China), followed by RT-qPCR amplification with TB Green^®^ Premix Ex Taq™ II (Tli RNaseH Plus; TaKaRa, Japan). We employed the 2^−∆∆CT^ method to determine the relative mRNA expression levels, with β-Actin serving as the internal control gene.

### 2.12. Statistical Analysis

Data are expressed as mean ± standard error (Mean ± SEM). Microsoft Excel and SPSS version 26.0 (IBM, Armonk, NY, USA) were used for statistical analysis. The Shapiro–Wilk test was performed using Graphpad Prism (v8) to ensure the data conformed to a normal distribution. Additionally, a Welch-corrected *t*-test was used to ensure significance even with unequal variances. Principal component analysis (PCA) and linear regression analysis were performed and plotted using an online tool (https://www.bioinformatics.com.cn/, last acccessed on 1st April 2025). Significance levels were defined as *p* < 0.05 *, *p* < 0.01 **, and *p* < 0.001 ***.

## 3. Results

### 3.1. Phenotypic Characteristics of Testicular Tissue Pre- and After Post-Maturity

**Figure 1:**
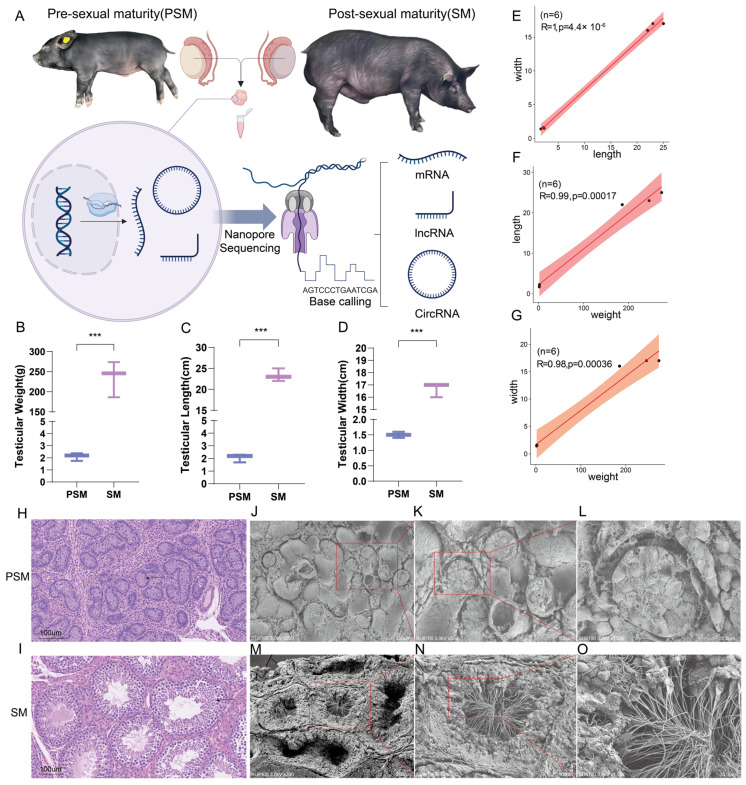
Experimental design, phenotypic characteristics, and histological and ultrastructural analysis of pigs before and after sexual maturity. (**A**): Schematic diagram of the experimental procedure. Pigs at pre-sexual maturity (PSM) and post-sexual maturity (SM) were selected as research subjects. After collecting samples, transcripts such as mRNA, lncRNA, and circRNA were detected by nanopore sequencing. (**B**–**D**): Phenotypic statistical bar charts of testicular weight, length, and width in the PSM and SM groups. (**E**–**G**): Scatter plots of correlations of testicular traits. (**E**) represents the correlation between testicular length and width, (**F**) represents the correlation between testicular weight and length, and (**G**) represents the correlation between body weight and body width. (**H**,**I**): HE-stained sections of tissues from the PSM and SM stages. The arrow points to the seminiferous tubule structure. (**J**–**L**): SEM of the ultrastructure from the PSM stages. (**M**–**O**): SEM of the ultrastructure from the SM stages. The lower right corner of the electron microscope image shows scale bars for 200 μm, 100 μm, and 30 μm, respectively. The red box indicates the seminiferous tubule structure. Significance levels were defined as *p* < 0.001 ***.

This study selected a crossbreed population of Tibetan pigs and Yorkshire pigs as the research subject. The overall experimental design was based on the Nanopore platform, which sequenced testicular tissues from two stages (PSM and SM) of the hybrid pigs and analyzed the expression patterns of three gene transcripts (Figure 1A). Phenotypic analysis of the pigs from the sample source revealed that, as representative time points before and after sexual maturity, the testicular weight of T × Y hybrid pigs at weaning was 2.110 ± 0.324 g, while that at the fattening stage was 235.43 ± 44.76 g. The weight, length, and width of the testes differed significantly before and after sexual maturity (*p* < 0.001). Length and width were proportional to weight, and length and width were also proportional to size before and after sexual maturity, which is consistent with the expected growth pattern (Figure 1B–G). Furthermore, HE sections showed that the testicular tissue at the PSM stage lacked complete seminiferous tubule structures, while the SM stage exhibited obvious seminiferous tubule structures (Figure 1H,I). SEM revealed that the testicular tissue at the PSM stage contained substantial precursor structures for seminiferous tubule formation, laying the structural foundation for spermatogenesis (Figure 1J–L). During the SM stage, the testes showed clear cross-sections of seminiferous tubules and sperm production (Figure 1M,N). These results indicate that the weaning period (28 days) and the fattening period (210 days) can serve as decisive time points for PSM and SM, providing phenotypic data to support subsequent research.

### 3.2. The mRNA Expression and Function Analysis of Testicular Tissues Pre- and Post-Sexual Maturity

**Figure 2:**
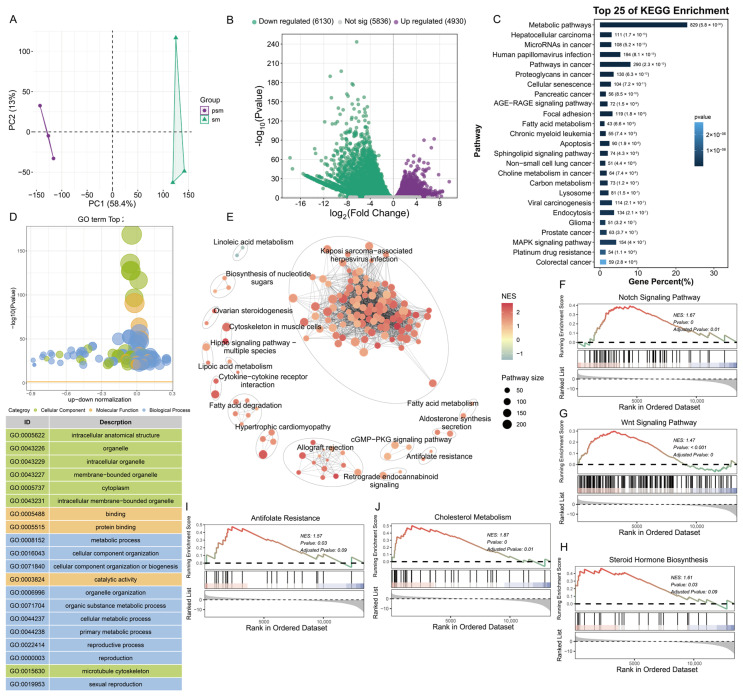
Differential expression characteristics and functional enrichment analysis of porcine mRNA before and after sexual maturity. (**A**): Principal component analysis (PCA) plot of transcripts from PSM and SM groups; (**B**): Volcano plot of differentially expressed transcripts, green represents downregulation, purple represents upregulation, and gray represents no significant difference; (**C**): Top 25 KEGG pathways enriched by differentially expressed transcripts; (**D**): GO functional enrichment bubble plot of differentially expressed transcripts, with functional classification including cellular components, molecular functions, and biological processes; (**E**): KEGG pathway association network diagram of differentially expressed transcripts, with nodes representing pathways and node size corresponding to the number of enriched genes; (**F**–**J**): GSEA enrichment analysis curves, showing the enrichment results of Notch signaling pathway, Wnt signaling pathway, steroid hormone synthesis, antifolate resistance, and cholesterol metabolism pathway in sequence (NES is the standardized enrichment score, and the color corresponds to the score).

To dissect the transcriptional dynamics underlying porcine testicular maturation and spermatogenesis, principal component analysis (PCA) of mRNA transcriptomes was conducted. The pre-sexual maturity (PSM) and post-sexual maturity (SM) groups were clearly separated along the PC1 (Figure 2A). This distinct clustering indicated profound transcriptional reprogramming during the transition from pre- to post-sexual maturity, laying a foundation for further analysis of genes related to testicular development. Differential expression analysis identified a total of 11,060 differentially expressed genes (DEGs), among which 4930 were upregulated and 6130 were downregulated in the SM group compared to the PSM group (Figure 2B).

Gene Ontology (GO) enrichment analysis, focusing on terms associated with testicular development and spermatogenesis, revealed targeted functional distributions of DEGs across three core categories. In the biological process (BP) category, DEGs were prominently enriched in reproductive processes (GO:0022414), spermatogenesis, and male gamete generation—terms that directly correspond to the initiation and progression of sperm production, a hallmark of testicular maturity. For the cellular component (CC) category, key enrichments included sperm flagellum and microtubule cytoskeleton, reflecting the structural remodeling of testicular cells (e.g., formation of mature sperm structures) essential for spermatogenesis. In the molecular function (MF) category, DEGs were enriched in protein binding and catalytic activity, which support the molecular interactions and enzymatic reactions required for regulating spermatogenic processes such as germ cell differentiation and sperm maturation (Figure 2C). KEGG enrichment analysis, prioritizing pathways linked to testicular function, further delineated key regulatory mechanisms. DEGs were significantly enriched in steroid hormone biosynthesis—a pathway critical for Leydig cell function and testosterone production, which is a central driver of spermatogenesis and testicular growth. Additionally, the MAPK signaling pathway (regulating testicular cell proliferation and differentiation during maturation), sphingolipid signaling pathway, and fatty acid metabolism (supporting the high energy demand for sperm meiosis and structural maturation) were also enriched, collectively underscoring the coordination of hormone synthesis and energy metabolism in testicular maturation (Figure 2D). GSEA further confirmed the enrichment of testicular development-related pathways in the SM group: steroid hormone biosynthesis (normalized enrichment score [NES] = 1.61, adjusted *p* = 0.09), Wnt signaling pathway (NES = 1.47, adjusted *p* < 0.001; regulating spermatogonial stem cell self-renewal and differentiation), and cytoskeleton in muscle cells (NES = 1.67, adjusted *p* = 0.01; supporting sperm flagellum assembly) (Figure 2E–J).

Collectively, these results demonstrate that mRNA transcriptional reprogramming during porcine testicular sexual maturity is dominated by DEGs enriched in spermatogenesis-related biological processes (e.g., male gamete generation) and testicular function-supporting pathways (e.g., steroid hormone biosynthesis, MAPK signaling). These transcriptional changes collectively drive the structural remodeling of testicular cells, functional maturation of the testis, and initiation of spermatogenesis, providing a molecular basis for understanding porcine male reproductive development.

### 3.3. The Transcript Expression and Function Analysis of Testicular Tissues Pre- and Post-Sexual Maturity

**Figure 3:**
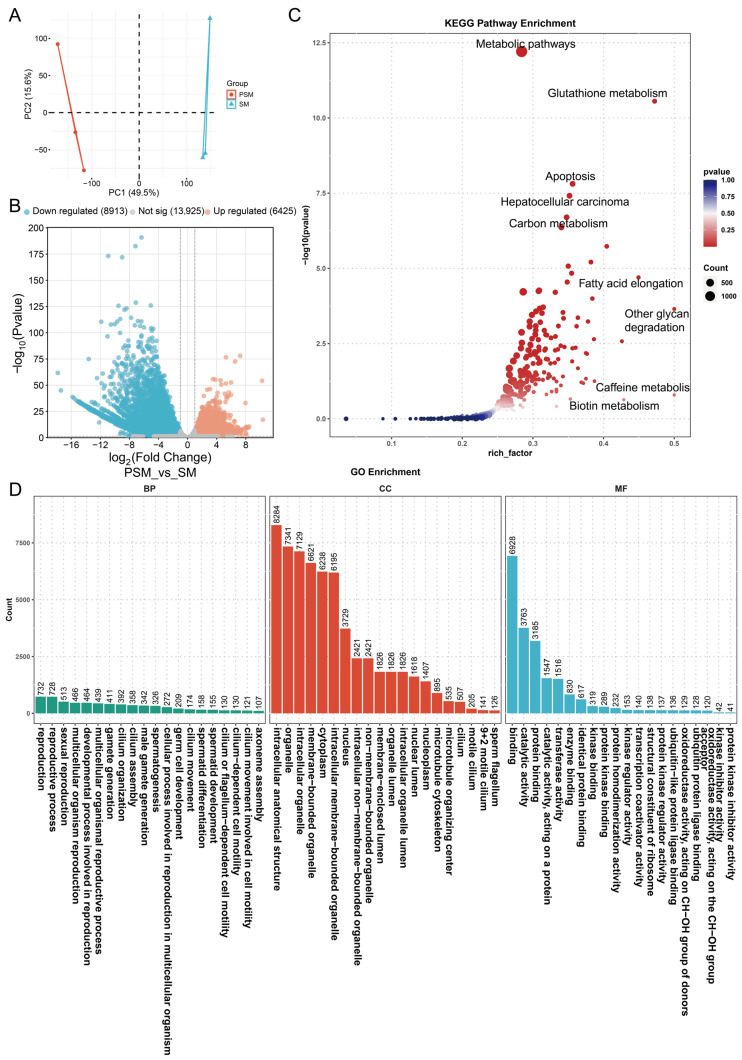
Principal component analysis and functional enrichment analysis of differentially expressed transcripts in pigs before and after sexual maturity. (**A**): Principal component analysis (PCA) plot of transcripts from the PSM and SM groups, with different colors representing the corresponding groups; (**B**): Volcano plot of differentially expressed transcripts, with blue representing downregulation, orange representing upregulation, and gray representing no significant difference; (**C**): Bubble plot of KEGG pathway enrichment; (**D**): Bar chart of GO functional enrichment.

To further decipher the transcriptional regulation of porcine testicular maturation at the transcript isoform level, we analyzed the expression dynamics of transcripts between the PSM and SM groups. Principal component analysis (PCA) of transcriptome data showed clear separation between the two groups along PC1 (49.5% of total variance) and PC2 (15.6% of total variance) (Figure 3A), indicating that sexual maturity induces distinct transcriptome remodeling at the isoform level, consistent with the gene-level transcriptional changes but reflecting more refined regulatory differences. Differential expression analysis identified 15,338 differentially expressed transcripts (DETs), including 6425 upregulated and 8913 downregulated transcripts in the SM group compared to the PSM group, with 13,925 transcripts showing no significant expression alteration (Figure 3B). The larger number of DETs than DEGs suggested that alternative splicing or transcript isoform switching might play crucial roles in regulating testicular maturation.

We used DETs to perform KEGG pathway enrichment analysis (Figure 3C) and found that the pathways with the highest enrichment were mainly composed of metabolic processes, including metabolic pathways (highest rich factor), glutathione metabolism, carbon metabolism, and fatty acid elongation. Additionally, pathways related to cell survival and function, such as apoptosis and hepatocellular carcinoma, were also enriched, implying that transcript isoform changes are involved in balancing cell proliferation, differentiation, and metabolic homeostasis during testicular maturation. The rich factor and −log10(*p* value) values indicated that these pathways are not only highly enriched but also statistically reliable. GO enrichment analysis further categorized the functional roles of DETs into BP, CC and MF (Figure 3D). For BP, DETs were most significantly enriched in reproduction-related processes, including sexual reproduction, multicellular organism reproduction, gamete generation, male gamete generation, and spermatogenesis, with spermatid differentiation and development also prominently represented. This highlighted that transcript-level regulation is tightly linked to the initiation and progression of spermatogenesis. For CC, key enriched terms included intracellular anatomical structures, membrane-bounded organelles, microtubule cytoskeleton, and sperm flagellum, which directly correspond to the structural maturation of seminiferous tubules and spermatozoa observed in phenotypic analysis. For MF, catalytic activity, protein binding, transferase activity, and kinase-related binding activities were the top enriched terms, reflecting enhanced enzymatic reactions and molecular interactions mediated by transcript isoforms to support testicular functional maturation.

### 3.4. LncRNA Expression Characteristics in Testicular Tissues Pre- and Post-Sexual Maturity

**Figure 4:**
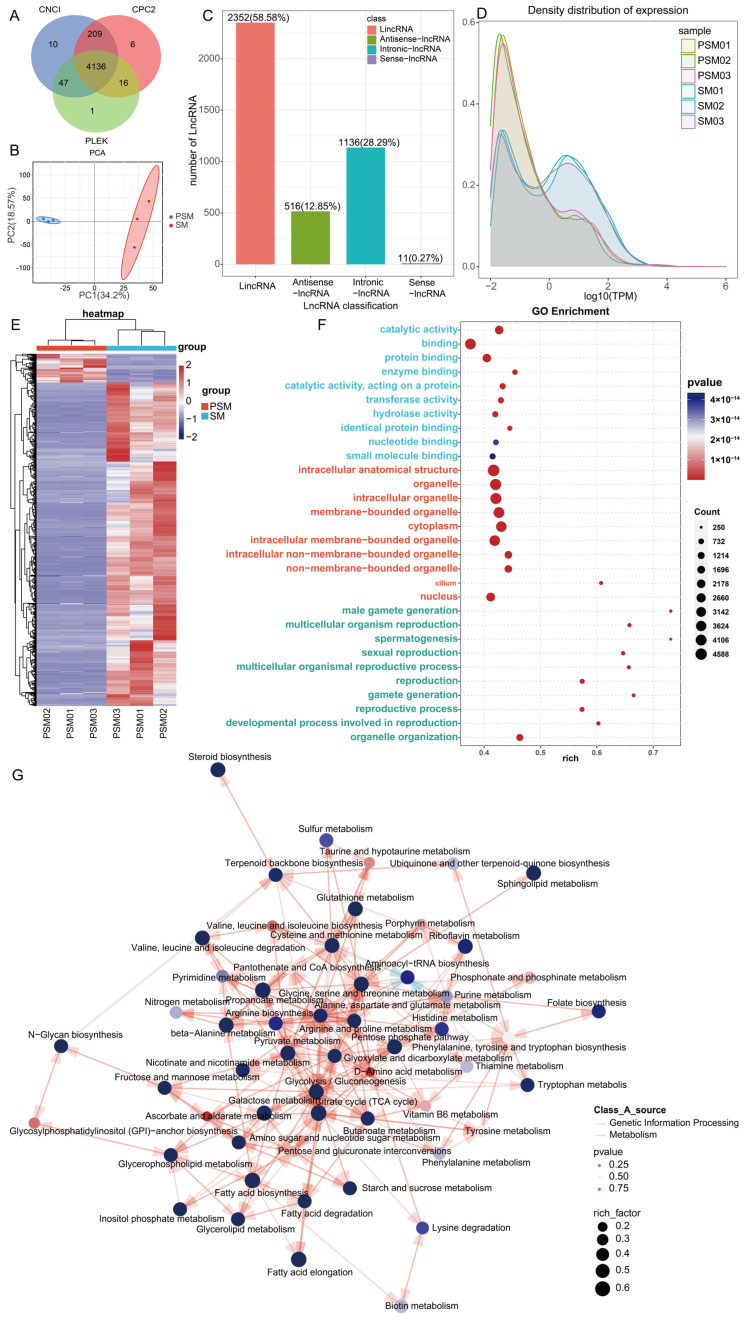
Characteristics and functional pathway enrichment analysis of porcine non-coding RNAs before and after sexual maturity. (**A**): Venn diagram of lncRNAs with ONCI, CRPC2, and PILEK (numbers indicate the number of overlapping transcripts); (**B**): PCA of the PSM and SM groups; (**C**): Bar chart of the number of different types of ncRNAs (lncRNA, Antisense-lncRNA, etc.) (with percentages indicated); (**D**): Density distribution curves of transcript expression levels in the PSM and SM groups; (**E**): Heatmap of differentially expressed transcripts in the PSM and SM groups (red and blue represent upregulation and downregulation, respectively); (**F**): GO functional enrichment bubble chart; (**G**): KEGG pathway enrichment network, annotating metabolic processes encompassing steroid biosynthesis and amino acid metabolism, with node parameters corresponding to enrichment characteristics.

To further decipher the regulatory roles of non-coding RNAs in porcine testicular maturation, we systematically analyzed the expression characteristics of lncRNAs in the PSM and SM groups based on Nanopore sequencing data. A total of 4136 lncRNAs were identified through combined prediction by CNCI, CPC2, and PLEK software, and classified into four categories according to their genomic locations (Figure 4A). Among these, long intergenic non-coding RNAs (LincRNAs) were the most abundant, accounting for 58.58% (2352) of the total lncRNAs, followed by intronic-lncRNAs (1136, 28.29%), antisense-lncRNAs (516, 12.85%), and sense-lncRNAs (11, 0.27%), indicating a predominant presence of intergenic lncRNAs in testicular tissues during sexual maturation. The number of lncRNAs detected in individual samples ranged from 1000 to 2000, with consistent distribution across PSM and SM groups, reflecting the stability of lncRNA identification (Figure 4A). PCA of lncRNA expression abundance showed separation between the PSM and SM groups along the first principal component (PC1, 34.2% of total variance) and the second principal component (PC2, 18.57% of total variance) (Figure 4B). This distinct clustering suggested that sexual maturity induces significant reprogramming of lncRNA expression patterns, which is consistent with the transcriptional changes observed at the mRNA and transcript levels. The density distribution of lncRNA expression levels (log10(TPM)) further revealed differences in expression abundance between the two groups, with the SM group showing a shifted distribution profile compared to the PSM group, indicating altered lncRNA expression intensity during testicular maturation (Figure 4C).

Differential expression analysis identified 688 differentially expressed lncRNAs (DELs) between the PSM and SM. To deduce the potential biological functions of DELs, we conducted functional enrichment analysis, with GO enrichment results grouped into BP, CC, MF (Figure 4D). For BP, DELs were significantly enriched in reproduction-related processes, including sexual reproduction, multicellular organism reproduction, gamete generation, male gamete generation, and spermatogenesis, highlighting the close association of lncRNA regulation with testicular reproductive function maturation. For CC, key enriched terms included intracellular anatomical structures, membrane-bounded organelles, cytoplasm, and nucleus, which align with the structural remodeling of testicular cells and seminiferous tubules during sexual maturity. For MF, the top enriched terms were catalytic activity, protein binding, transferase activity, and hydrolase activity, suggesting that DELs may regulate testicular development by mediating enzymatic reactions and molecular interactions. KEGG pathway enrichment analysis showed that DELs were primarily enriched in metabolic pathways (Figure 4E). The top enriched pathways included steroid biosynthesis, sulfur metabolism, taurine and hypotaurine metabolism, terpenoid backbone biosynthesis, and N-glycan biosynthesis, which is consistent with the abstract’s mention of lncRNAs being linked to N-glycan biosynthesis. Additionally, pathways such as glutathione metabolism, cysteine and methionine metabolism, and fatty acid metabolism were also significantly enriched, indicating that lncRNAs may participate in regulating energy metabolism, hormone synthesis, and redox homeostasis during testicular maturation. The high rich factor values of these pathways confirmed their strong correlation with DEL expression changes.

### 3.5. Detection and Differences in circRNA in Testicular Tissues Before and After Sexual Maturity

**Figure 5:**
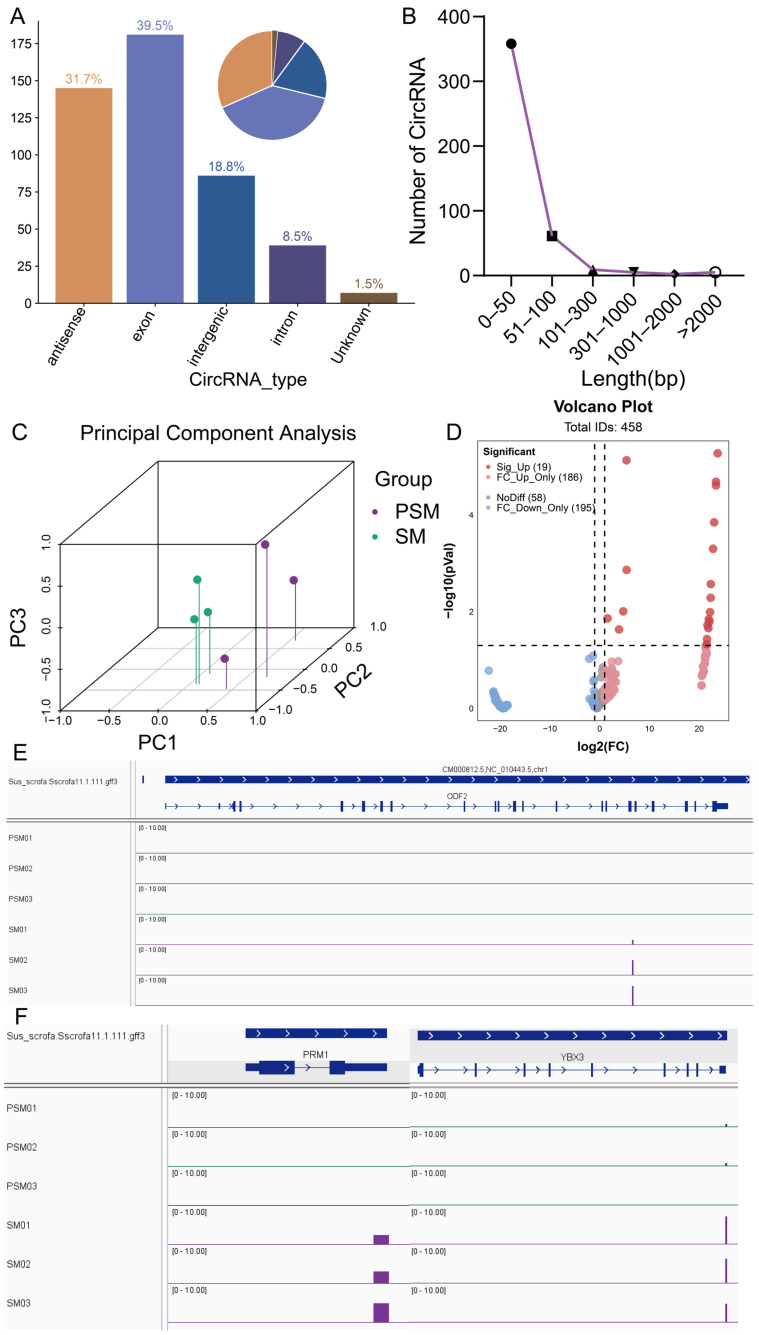
Characteristic analysis of porcine circRNAs before and after sexual maturity and visualization of IGV of key reproduction-related genes. (**A**): Statistical analysis of circRNA types (bar chart on the left, pie chart on the right); (**B**): Length distribution of circRNAs; (**C**): Three-dimensional principal component analysis (PCA) of circRNA expression profiles in PSM and SM groups, with different colors representing corresponding groups; (**D**): Volcano plot of differential expression of circRNAs, marking categories such as significantly upregulated (Sig. Up) and significantly downregulated (Sig. Down), where dashed lines indicate differential analysis thresholds set to *p* < 0.05 and |log2(FC)| > 1.; (**E**,**F**): IGV visualization of key genes, with (**E**) showing the expression signal of *ODF2* and (**F**) showing the expression signals of *PRM1* and *YBX3*. Arrows indicate the direction of transcription.

The circRNAs play crucial regulatory roles in various biological processes. To explore the potential functions of circRNAs in porcine testicular maturation, we systematically identified and analyzed circRNAs in PSM and SM groups using CIRI-Long software (v1.1.0) based on Nanopore sequencing data. A total of 458 circRNA transcripts were successfully detected, with distinct length distribution characteristics (Figure 5A). Among these, the largest proportion (39.5%) of circRNAs fell within a specific length range, followed by 31.7%, 18.8%, 8.5%, and 1.5% in other length intervals, indicating a relatively concentrated length distribution of testicular circRNAs, which may be related to their structural stability and functional specificity. PCA of circRNA expression profiles showed clear separation between the PSM and SM groups (Figure 5C), demonstrating that the circRNA expression pattern undergoes significant remodeling during the transition from pre- to post-sexual maturity, which is consistent with the transcriptional reprogramming observed in mRNAs, transcripts, and lncRNAs. Classification statistics of circRNA expression differences (Figure 5D) revealed that among the 458 circRNAs, 19 were significantly differentially expressed, which was consistent with the result summarized in the abstract. These differentially expressed circRNAs (DEcircRNAs) were predominantly upregulated (Sig_Up = 19), with no significantly downregulated circRNAs detected. Additionally, 186 circRNAs showed fold change upregulation only (FC_Up_Only), 195 showed fold change downregulation only (FC_Down_Only), and 58 showed no significant expression difference (NoDiff), further confirming that sexual maturity is accompanied by widespread changes in circRNA expression, with upregulation as the main trend. IGV visualization of circRNA location and expression abundance (Figure 5E,F). For example, circRNAs(chr1_268886055–268886097) in *ODF2* showed distinct expression differences between the PSM and SM groups, with significantly higher expression levels in the SM group (Figure 5E). Additionally, circRNAs (chr3_31861469–31861512) in *PRM1* and circRNA(chr5_61361204–61361253) in *YBX3* also exhibited group-specific expression patterns (Figure 5F), which were closely associated with the initiation and progression of spermatogenesis in mature testes. These results suggested that DEcircRNAs may participate in regulating testicular functional maturation by targeting key genes involved in spermatogenesis.

### 3.6. Combined Analysis of Multiple Transcribed RNAs Elucidates the Synergistic Expression of Genes During Porcine Testicular Development

**Figure 6:**
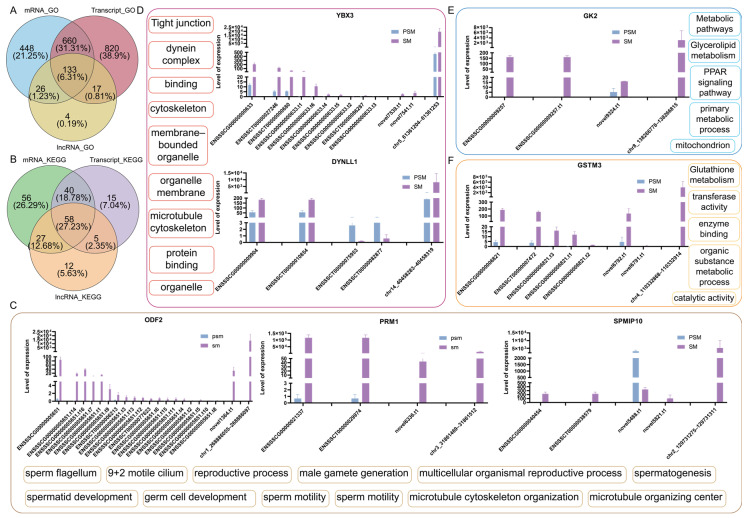
Functional enrichment characteristics of porcine mRNA/transcript/lncRNA/circRNA before and after sexual maturity, and expression and functional associations of key genes. (**A**): Venn diagram of common GO pathways for mRNA, transcript, and lncRNA; (**B**): Venn diagram of common KEGG pathways for mRNA, transcript, and lncRNA; (**C**): Expression abundance of *ODF2*, *PRM1*, and *SPMIP10* genes in the four transcripts and common enrichment pathways; (**D**): Expression abundance of *YBX3* and *DYNLL1* genes in the four transcripts and common enrichment pathways; (**E**): Expression abundance of *GK2* gene in the four transcripts and common enrichment pathways; (**F**): Expression abundance of *GSTM3* gene in the four transcripts and common enrichment pathways.

To decipher the coordinated coding and ncRNA regulatory circuitry in porcine testicular maturation, we integrated the analysis of mRNAs, transcripts, lncRNAs, and circRNAs, focusing on shared functional enrichment pathways and their corresponding core genes. GO enrichment Venn diagram analysis (Figure 6A) identified 133 commonly enriched GO terms across mRNAs, transcripts, and lncRNAs, accounting for 6.31% of the total enriched terms. KEGG enrichment Venn diagram analysis (Figure 6B) identified 58 commonly enriched pathways across mRNAs, transcripts, and lncRNAs, all of which harbor the core genes analyzed in this study.

We additionally examined the expression levels of core genes across four RNA types: gene, transcript, lncRNA-cistarget, and circRNA (Figure 6C–F). All four RNA types showed consistent expression trends between the PSM and SM groups, with coordinated upregulation in mature testes, reflecting synergistic regulation of core pathways. For sperm structure and chromatin condensation-related core genes (Figure 6C), *ODF2*, *PRM1*, and *SPMIP10* showed minimal expression in the PSM group across all four RNA types. In the SM group, the gene, transcript, lncRNA-cistarget, and circRNA levels were dramatically enhanced with consistent expression patterns, validating their synergistic role in sperm formation and maturation through shared functional pathways. For spermatogenesis and cytoskeleton-related core genes (Figure 6D), *YBX3* and *DYNLL1* exhibited low expression in the PSM group across all four RNA types. In the SM group, the expression abundance of the corresponding gene, transcript, lncRNA-cistarget, and circRNA was significantly upregulated, consistent with the activation of spermatogenesis and structural maturation of sperm flagellum in mature testes. For energy metabolism-related core gene (Figure 6E), *GK2* showed low expression in the PSM group across all four RNA types. In the SM group, the expression levels of the gene, transcript, lncRNA-cistarget, and circRNA were synergistically elevated, supporting the enhanced energy supply required for testicular maturation and spermatogenesis via shared metabolic pathways. For redox balance-related core gene (Figure 6F), *GSTM3* exhibited low expression in the PSM group across all four RNA types. In the SM group, the gene, transcript, lncRNA-cistarget, and circRNA levels were sharply upregulated, maintaining cellular redox homeostasis during testicular structural remodeling and spermatogenesis through the shared KEGG pathway.

### 3.7. RT-qPCR Was Used to Validate the Expression Patterns of Core Functional Genes and Spermatogenesis-Related Genes in Nanopore DRS

**Figure 7:**
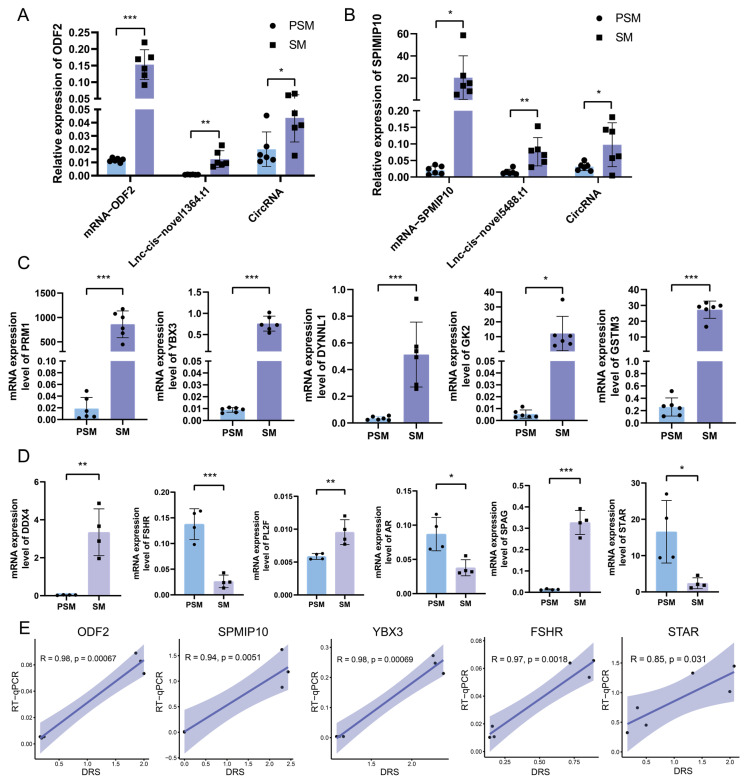
Validation of differentially expressed genes by RT-qPCR and correlation analysis with Nanopore DRS data. (**A**,**B**): Relative expression abundance of mRNAs, lncRNAs, and circRNAs associated with *ODF2* and *SPMIP10*. (**C**): Relative mRNA expression levels of core functional genes (*PRM1*, *YBX3*, *DYNLL1*, *GK2*, *GSTM3*) in PSM and SM groups detected by qRT-PCR. (**D**): Relative mRNA expression levels of key reproductive marker genes (*DDX4*, *STAR*, *SPAG*, *AR*, *FSHR*, *PL2F*) in PSM and SM groups. (**E**): Correlation scatter plots between qRT-PCR results and Nanopore DRS (Direct RNA Sequencing) data for *YBX3*, *FSHR*, *ODF2*, *SPMIP10*, and *STAR*. Significance levels were defined as *p* < 0.05 *, *p* < 0.01 **, and *p* < 0.001 ***.

To verify the reliability of the transcriptional expression patterns obtained from nanopore DRS, we performed qRT-PCR analysis on 13 key genes, including 7 core functional genes and 6 specific reproductive marker genes identified in the joint analysis. The expression trends of core genes (*ODF2*, *SPMIP10*, *PRM1*, *YBX3*, *DYNLL1*, *GK2*, *GSTM3*) in the PSM and SM groups were completely consistent with the nanopore sequencing results. Specifically, taking *ODF2* and *SPMIP10* as examples, we verified the RT-qPCR fluorescence quantitative levels of mRNA, lncRNA, and circRNA in PSM and SM (Figure 7A,B). In addition, all core genes showed extremely low expression levels in the PSM group, while their mRNA levels were significantly upregulated in the SM group (Figure 7A–C). This reflects the synergistic activation of genes related to sperm structural maturation, spermatogenesis, energy supply, and redox homeostasis during testicular sexual maturation. For reproductive marker genes (Figure 7D), *DDX4*, *STAR*, *SPAG*, *AR*, *FSHR* and *PL2F* all showed significant differential expression between the two groups. Specifically, *STAR*, *SPAG*, *AR*, and *FSHR* exhibited significant upregulation in the SM group (*p* < 0.001), while *DDX4* and *PL2F* displayed strong expression specificity in mature testicular tissue—aligning with the activation of the hypothalamus–pituitary–gonadal axis and the onset of spermatogenesis during the SM stage. Correlation analysis between RT-qPCR and Nanopore DRS data (Figure 7E) showed that the relative expression levels of *YBX3* (R = 0.94, *p* = 0.0051), *FSHR* (R = 0.98, *p* = 0.00067), *ODF2* (R = 0.85, *p* = 0.031), *SPMIP10* (R = 0.98, *p* = 0.00069), and *STAR* (R = 0.97, *p* = 0.0018) were significantly positively correlated between the two detection methods. The high correlation coefficients and significant statistical significance confirm the accuracy and reliability of the Nanopore sequencing data in this study, providing strong experimental support for previous analysis of the transcriptional regulatory network during porcine testicular sexual maturation.

## 4. Discussion

The comprehensive transcriptional profiling of porcine testes before and after sexual maturity revealed extensive reprogramming of coding and non-coding RNAs, coupled with dramatic phenotypic remodeling. These results are not accidental but arise from the coordinated interplay of neuroendocrine regulation, cellular differentiation, and metabolic adaptation during testicular maturation [35]. The phenotypic transformation of testes—from small, structurally immature tissues with incomplete seminiferous tubules to large, functional organs capable of spermatogenesis—directly stems from the activation of the HPG at sexual maturity [36]. The weaning period (28 days) represents a pre-pubertal stage where gonocytes are differentiating into spermatogonial stem cells, and Sertoli/Leydig cells are in a quiescent state [37], explaining the minimal testicular weight (2.110 ± 0.324 g) and underdeveloped tissue structure. In contrast, the fattening period (210 days) coincides with HPG axis activation: gonadotropins (FSH and LH) stimulate Sertoli cells to cease proliferation and form the blood-testis barrier, while Leydig cells mature to secrete copious testosterone [14,38]—key signals that trigger spermatogenesis initiation and testicular growth (235.43 ± 44.76 g). This hormonal shift is the primary driver of the profound transcriptional reprogramming observed in mRNAs and transcripts, as genes involved in cell differentiation, structural formation, and reproductive function are activated, while genes associated with embryonic development and cell proliferation are downregulated.

The extensive differential expression of mRNAs (11,060 DEGs) and transcripts (15,338 DETs) reflects the functional specialization of testicular cells during maturation. The enrichment of DEGs in metabolic pathways (e.g., fatty acid metabolism, carbon metabolism) arises from the high energy demand of spermatogenesis—sperm production requires substantial ATP for meiosis, chromatin condensation, and flagellum assembly, necessitating enhanced nutrient catabolism and lipid synthesis [39]. The prominence of reproductive process-related GO terms (e.g., spermatogenesis, male gamete generation) is a direct consequence of spermatogenic lineage progression: spermatogonia develop into spermatocytes, progress through meiosis to become spermatids, and ultimately mature into spermatozoa, a process requiring the coordinated activation of genes such as *PRM1* [40] (chromatin condensation) and *ODF2* [41] (sperm outer dense fiber formation). Notably, DETs outnumber DEGs, suggesting that alternative splicing is pivotal for refining gene function during maturation—distinct transcript isoforms derived from a single gene are capable of regulating unique cellular events (e.g., proliferation vs. differentiation), allowing precise regulation of spermatogenesis without altering overall gene expression levels. For example, splice variants of cytoskeleton-related genes may adapt to the structural needs of sperm flagellum assembly, while metabolic enzyme isoforms optimize energy production for mature testicular cells [39,42].

The distinct expression characteristics of lncRNAs and circRNAs are shaped by their specialized regulatory roles in testicular maturation. The predominance of LincRNAs (58.58% of total lncRNAs) is likely due to their ability to act as trans-regulatory molecules, coordinating the expression of scattered spermatogenesis-related genes across the genome—essential for orchestrating the complex gene networks underlying cell differentiation. The 688 DELs enriched in steroid biosynthesis and N-glycan biosynthesis pathways reflect their involvement in hormone production and protein modification: lncRNAs may regulate Leydig cell steroidogenesis by modulating key enzymes (e.g., cytochrome P450 family members) [43,44] or facilitate sperm surface protein glycosylation (critical for sperm-egg recognition) via N-glycan biosynthesis [45]. For circRNAs, the detection of 458 transcripts with a concentrated length distribution arises from their biogenesis mechanism—circRNAs are generated by back-splicing of pre-mRNAs, and their stable, closed-loop structure makes them ideal for long-term regulatory roles in mature tissues. The exclusive upregulation of 19 DEcircRNAs post-puberty suggests they function as “maturation stabilizers”: by acting as miRNA sponges, they may sequester miRNAs that repress spermatogenesis-related genes (e.g., *YBX3*, *PRM1*), guaranteeing persistent expression of these genes throughout spermatogenesis.

The synergistic expression of core genes across four RNA types (gene, transcript, lncRNA-cistarget, circRNA) and their convergence on 133 shared GO terms and 58 KEGG pathways underscores the need for multi-layered regulation to ensure testicular maturation precision. This coordination arises from the complexity of spermatogenesis, which requires strict temporal and spatial control of gene expression [46]. For example, *GSTM3* (a key gene in glutathione metabolism) is synergistically upregulated across all RNA types to maintain redox homeostasis—reactive oxygen species (ROS) accumulate during spermatogenesis, and glutathione-dependent detoxification is essential to prevent sperm DNA damage [47,48]. Similarly, *GK2* (glycerolipid metabolism) and *DYNLL1* (microtubule dynamics) are co-regulated to meet energy demands and support sperm flagellum assembly, respectively. LncRNA-cistargets likely enhance transcription of these core genes via cis-regulatory interactions (e.g., binding to promoters or enhancers), while circRNAs stabilize their expression by inhibiting miRNA-mediated degradation. This multi-dimensional regulatory network minimizes noise and ensures robust execution of testicular maturation programs, avoiding aberrant gene expression that could impair reproductive function.

The enrichment of key signaling pathways (e.g., Wnt, MAPK, PI3K-Akt) in DEGs and DETs reflects their conserved roles in testicular development. The Wnt signaling pathway, enriched in the SM group, regulates Sertoli cell proliferation and spermatogonial stem cell self-renewal [49,50]—its activation at sexual maturity balances stem cell maintenance with differentiation to sustain continuous spermatogenesis. The MAPK pathway, involved in cell proliferation and differentiation, mediates testosterone-induced signaling in Leydig cells, further amplifying the reproductive program [51]. These pathways are not isolated but interact synergistically: for instance, PI3K-Akt signaling enhances metabolic activity to support MAPK-driven cell differentiation, while Wnt pathway activation modulates the expression of metabolic enzymes [52,53]. This crosstalk ensures that cellular differentiation, structural formation, and metabolic adaptation proceed in a coordinated manner, explaining the convergence of multiple RNA types on these core pathways. the transcriptional and phenotypic changes observed in porcine testes during sexual maturity are driven by HPG axis activation, which triggers a cascade of hormonal signals that rewire the testicular transcriptome. Coding RNAs provide the functional backbone for spermatogenesis and metabolic adaptation, while alternative splicing (transcripts) and non-coding RNAs (lncRNAs, circRNAs) refine regulatory precision. The synergistic coordination of these RNA types ensures the orderly progression of cellular differentiation, structural remodeling, and metabolic activation—key processes that enable the testes to acquire reproductive competence. These findings deepen our understanding of the molecular mechanisms governing mammalian male reproduction and highlight the complexity of multi-layered transcriptional regulation in organ maturation.

It is important to acknowledge the limitations of the present study to provide a balanced perspective for future research. First, regarding the sample size, this study employed six F1 hybrid pigs (3 per group) due to the high cost of Oxford Nanopore Direct RNA Sequencing and adherence to the 3Rs principle of animal experimentation. Although Welch’s t-test was used to correct for variance heterogeneity and qRT-PCR validation confirmed the reliability of key findings, expanding the sample size to include more individuals with diverse genetic backgrounds and production environments will be essential to verify the robustness of the identified transcriptional regulatory networks in future studies. Second, as this research used domestic pigs as the animal model, inherent biological differences between pigs and humans—including variations in genetic regulatory elements, hypothalamic–pituitary–gonadal axis modulation, and spermatogenic developmental kinetics—must be considered when extrapolating the conclusions to human reproductive biology. While pigs share significant physiological and anatomical similarities with humans, the specific roles of the identified core genes (e.g., PRM1, ODF2, GSTM3) and non-coding RNA regulatory networks in human testicular maturation require further validation through cross-species comparative studies or clinical translational research. Additionally, this study focused on two discrete time points (pre-sexual maturity at 28 days and post-sexual maturity at 210 days), which may not capture the dynamic transcriptional changes during the intermediate stages of testicular development; future studies incorporating a time-series design could provide a more comprehensive understanding of the sequential regulatory mechanisms underlying sexual maturation. These limitations not only highlight the need for caution in interpreting the broader applicability of current research findings, but also provide valuable directions for refining and expanding the research to enhance its translational potential in animal genetics and reproduction, as well as human reproductive medicine.

## 5. Conclusions

This study analyzed porcine testicular transcriptomes at pre-sexual maturity (PSM, 28 days) and post-sexual maturity (SM, 210 days) by Oxford Nanopore long-read sequencing. SM testes showed marked growth (2.110 ± 0.324 g to 235.43 ± 44.76 g) with complete seminiferous tubules and spermatogenesis initiation, along with 11,060 DEGs, 15,338 DETs, 688 DELs, and 19 DEcircRNAs enriched in spermatogenesis-related processes/pathways. A key finding was the synergistic upregulation of seven core genes (*ODF2*, *PRM1*, *SPMIP10*, *YBX3*, *DYNLL1*, *GK2*, *GSTM3*) across gene, transcript, lncRNA-cistarget, and circRNA levels in SM testes—*ODF2* and *PRM1* ensure sperm structural integrity and functional competence, SPMIP10 and *YBX3* drive spermatogenic progression, *DYNLL1* supports cytoskeletal remodeling of seminiferous tubules, *GK2* meets the high energy demand of spermatogenesis via glycerolipid metabolism, and *GSTM3* maintains cellular redox balance. Additionally, long intergenic non-coding RNAs (LincRNAs) dominated lncRNA subtypes to coordinate spermatogenesis-related gene expression, DELs participated in steroid and N-glycan biosynthesis, and SM-upregulated DEcircRNAs likely stabilized spermatogenic gene expression. This work fills a gap in porcine testicular long-read transcriptome research, elucidating a multi-RNA co-regulatory network centered on seven core genes. These core genes and differentially expressed RNAs can serve as valuable molecular markers for early screening of high-fertility boars in pig breeding, helping to predict semen quality and reduce feeding costs. Enriched pathways, such as steroid biosynthesis pathways, provide targets for nutritional regulation or genetic modification to accelerate sexual maturation and increase semen production in livestock. Furthermore, constructing a multi-RNA co-regulatory network for porcine testicular sexual maturation directly links basic research with breeding production and biomedicine, and provides high-resolution genomic resources to advance the development of mammalian reproductive biology.

## Figures and Tables

**Table 1:** Data quality and mapping rate statistics of Direct RNA sequences based on the Nanopore platform. TotalReads is the total number of reads obtained from sequencing. MaxLen is the length of the longest read in bp. AvgLen is the average length of the reads in bp. meanQ is the quality score. MappedReads is the number of reads that were aligned to the reference genome Sus scrofa11.1; MapRate is the alignment rate statistics.

Sample	TotalReads	MaxLen	AvgLen	meanQ	MappedReads	MapRate
PSM_1	4,133,792	466,657	1261.13	19.12	3,961,589	95.83%
PSM_2	4,018,445	345,297	1266.93	19.56	3,867,480	96.24%
PSM_3	3,793,142	402,379	1269.08	19.86	3,653,531	96.32%
SM_1	3,551,651	387,907	1287.94	19.86	3,450,932	97.16%
SM_2	4,153,072	397,724	1099.70	20.64	4,002,856	96.38%
SM_3	4,547,085	323,717	1058.91	20.5	4,379,263	96.31%

## Data Availability

All data involved in this study can be obtained from the corresponding authors.

## Abbreviations

The following abbreviations are used in this manuscript:

LncRNA	Long Non-Coding RNA
mRNA	messenger RNA
CircRNA	Circular RNA
DRS	Direct RNA Sequencing
PSM	Pre-Sexual Maturity
SM	Post-Sexual Maturity
CDS	Coding Sequences
ORF	Open Reading Frame
HPG	hypothalamic–pituitary–gonadal axis
